# Inflammation, Organ Failure, and the Path to Surgery in Acute Pancreatitis

**DOI:** 10.3390/medicina62020349

**Published:** 2026-02-09

**Authors:** Oprescu Macovei Anca Monica, Dana Paula Venter, Stefan Mihai, Constantin Oprescu, Andrei Gabriel, Dumitriu Bogdan, Micle Bianca-Maria, Valcea Sebastian, Gheorghiu Alexandra-Oana, Ilie Madalina

**Affiliations:** 1 Faculty of Medicine, University of Medicine and Pharmacy Carol Davila, 050474 Bucharest, Romaniaandrei-gabriel.nicolae@umfcd.ro (A.G.); bogdan.dumitriu@drd.umfcd.ro (D.B.);; 2 Gastroenterology Department, Emergency Clinical Hospital, Agrippa Ionescu, 011356 Bucharest, Romania; 3 Pediatrics Surgery Department, Emergency Clinical Hospital, Grigore Alexandrescu, 011743 Bucharest, Romania; 4 General Surgery Department, Emergency Clinical Hospital, Floreasca, 014461 Bucharest, Romania; 5 Gastroenterology Department, Emergency Clinical Hospital, Floreasca, 014461 Bucharest, Romania

**Keywords:** acute pancreatitis, IL-6, necrotizing pancreatitis, infected necrosis, organ failure, predictive model, surgery, biomarkers

## Abstract

*Background and Objectives:* Early identification of patients with acute pancreatitis (AP) who will require operative intervention remains a major clinical challenge. Traditional severity scores and delayed inflammatory biomarkers offer limited early predictive accuracy. This study aimed to evaluate the combined predictive value of IL-6, necrotic burden, and organ failure parameters for determining the need for surgical management in AP. *Materials and Methods:* We performed a retrospective cohort study of 325 consecutive patients with AP admitted to Floreasca Emergency Hospital between January 2020 and December 2024. Clinical, laboratory, and imaging parameters were collected, including IL-6 at 24 h, CRP at 48–72 h, SOFA and BISAP scores, and morphologic complications. The primary endpoint was the requirement for operative intervention. Univariate and multivariable logistic regression analyses were conducted, and model performance was assessed using ROC curves and calibration testing. *Results:* Sixty patients (18.5%) underwent surgery for pancreatitis-related complications. Operated patients demonstrated higher IL-6 levels (median 120 vs. 55 pg/mL), necrosis >30% (68.3% vs. 14%), infected necrosis (55% vs. 2.3%), and persistent organ failure (46.7% vs. 9.8%) (all *p* < 0.001). In multivariable analysis, IL-6 at 24 h (OR 1.35 per 50 pg/mL), necrosis > 30% (OR 4.80), infected necrosis (OR 6.20), persistent organ failure ≥48 h (OR 2.60), SOFA score (OR 1.18 per point), and CRP at 48–72 h (OR 1.09 per 50 mg/L) independently predicted surgery. The final model showed excellent discrimination (AUC 0.88) and good calibration. *Conclusions:* IL-6-driven inflammatory escalation, combined with necrotic burden and persistent organ dysfunction, provides a robust early predictive framework for identifying AP patients likely to require surgical intervention. Integration of these parameters may improve triage, timing of intervention, and multidisciplinary decision-making in acute pancreatitis.

## 1. Introduction

Acute pancreatitis (AP) is an inflammatory disease with a highly heterogeneous clinical course, ranging from mild, self-limited episodes to severe necrotizing forms complicated by multiorgan failure. According to the Revised Atlanta Classification—developed through international consensus by Banks et al. (2013; expert working group review) [1]—approximately 15–20% of patients progress to moderately severe or severe AP, a subgroup associated with increased morbidity, mortality, and prolonged hospitalization. Early recognition of patients at risk for deterioration remains essential for guiding appropriate monitoring intensity, ICU allocation, and multidisciplinary management.

Over the past decade, numerous studies have evaluated early predictors of severe AP, including clinical scores (BISAP, SOFA), systemic inflammatory response syndrome (SIRS), and biochemical markers such as C-reactive protein (CRP) and interleukin-6 (IL-6). Among these, IL-6 has consistently emerged as a promising early biomarker. Li et al. (2022; N = 328; retrospective cohort) [2] demonstrated that IL-6 surpassed CRP in predicting infected pancreatic necrosis (IPN) and mortality, while Wiese et al. (2022; N = 245; prospective observational study) [3] identified IL-6 as one of the earliest discriminators of patients who subsequently developed infected necrosis. Earlier work by Dambrauskas et al. (2006; N = 72; prospective clinical study) [4] similarly showed that cytokine activation, particularly IL-6, closely reflects the transition from sterile necrosis to infected disease.

Furthermore, recent predictive models have reinforced the value of early inflammatory markers. Mao et al. (2023; N = 311; prospective cohort) [5] reported that IL-6-integrated PASS and mPASS scores provided superior early prediction of necrotizing complications compared with CRP-based approaches. Across these studies, IL-6 consistently rises earlier and correlates more strongly with necrosis, persistent organ failure, and mortality than traditional markers, making it an attractive candidate for early risk stratification.

Despite this progress in predicting severity, there remains a notable gap in predicting which patients will ultimately require operative intervention. Contemporary surgical management, as shown in the randomized controlled trial by van Santvoort et al. (2010; N = 88; RCT) [6], is typically reserved for key complications such as infected necrosis, abdominal compartment syndrome, hemorrhage, or persistent organ failure despite maximal conservative therapy. While morphologic features—such as the extent of necrosis and the presence of infection—are known to influence operative decision-making, previous studies have rarely integrated these structural variables with early biochemical and organ-failure metrics into a unified predictive framework.

As a result, clinicians often lack robust early tools to anticipate which patients will progress toward invasive management. In our experience, the early clinical trajectory is shaped by interacting domains: (1) systemic inflammation (reflected by IL-6 and CRP), (2) physiologic deterioration (quantified by SOFA score and persistent organ failure), and (3) morphologic severity (extent of necrosis and infection status). These complementary dimensions likely represent the full spectrum of biological processes that drive progression from inflammation to complications requiring surgical intervention. However, these factors have rarely been evaluated simultaneously within a large, well-characterized clinical cohort.

The objective of this study was therefore to identify early independent predictors of surgical intervention by integrating inflammatory biomarkers, organ dysfunction scores, and morphologic severity indicators. Using a five-year cohort of 325 patients treated at a tertiary hepatopancreatobiliary center, we aimed to develop and validate a multivariable model capable of discriminating which patients are most likely to require operative management. We hypothesized that early IL-6 elevation, extensive or infected necrosis, and persistent organ failure would emerge as principal determinants of surgical need. By refining early risk assessment, our study seeks to provide clinicians with a more accurate framework for triage, multidisciplinary planning, and timely decision-making in acute pancreatitis.

## 2. Materials and Methods

This retrospective observational cohort study was conducted at the Department of General Surgery and Gastroenterology of Floreasca Emergency Hospital in Bucharest, Romania, a tertiary referral center specializing in hepatopancreatobiliary emergencies. The study included all consecutive adult patients admitted with acute pancreatitis between 1 January 2020 and 31 December 2024. Ethical approval was obtained from the Institutional Review Board of Floreasca Emergency Hospital (Approval No. 124/10.01.2020). All procedures adhered to the principles of the Declaration of Helsinki, and because the study was retrospective and all data were anonymized, informed consent was waived.

Length of hospital stay (LOS) was defined as the number of days from hospital admission to discharge or in-hospital death.

In-hospital mortality was defined as death occurring during the index hospitalization for acute pancreatitis.

Patients were considered eligible if they met the Revised Atlanta Classification criteria for acute pancreatitis, which require at least two of the following: characteristic epigastric pain, serum amylase or lipase levels at least three times the upper limit of normal, or imaging findings compatible with acute pancreatitis. Individuals with chronic pancreatitis, pancreatic malignancy, recurrent episodes of acute pancreatitis, incomplete clinical or imaging data, or restricted access to medical records were excluded. Clinical, laboratory, and imaging data were extracted from electronic medical records, laboratory databases, and radiology archives. Two independent investigators verified all variables to ensure accuracy.

Management decisions were made within a multidisciplinary framework involving gastroenterology, interventional radiology, surgery, and intensive care. Endoscopic and radiologic interventions were considered whenever technically feasible and clinically appropriate. The choice of primary surgical intervention was reserved for patients with life-threatening complications, unfavorable anatomy for minimally invasive access, or rapid clinical deterioration precluding delayed step-up approaches.

The collected dataset included demographic characteristics such as age, sex, body mass index, ASA physical status, smoking status, and alcohol consumption. Etiologic factors were documented, including gallstone disease, alcohol-related pancreatitis, hypertriglyceridemia, post-ERCP pancreatitis, and idiopathic cases. Disease severity was assessed using the BISAP score, the SOFA score, and the Revised Atlanta Classification. Inflammatory biomarkers were collected at standardized intervals, with interleukin-6 measured 24 h after admission and C-reactive protein measured between 48 and 72 h. Morphologic and clinical complications were assessed using contrast-enhanced imaging and clinical evaluation and included the percentage of pancreatic necrosis, the presence of infected necrosis, walled-off necrosis, persistent organ failure lasting at least 48 h, abdominal compartment syndrome, gastrointestinal or peripancreatic hemorrhage, and persistent biliary obstruction.

Timing of contrast-enhanced CT: Contrast-enhanced computed tomography (CECT) was performed according to institutional protocol and international recommendations. Routine CECT was not performed within the first 72 h after admission, unless clinical deterioration or diagnostic uncertainty mandated earlier imaging. In most patients, CECT was obtained after 72 h, once pancreatic necrosis was expected to be fully declared.

The median time from admission to first CECT was 4 days (interquartile range 3–6 days).

Operative outcomes were also documented, including whether a surgical intervention was required and the indication for that intervention, such as infected necrosis requiring debridement, abdominal compartment syndrome requiring decompression, hemorrhage requiring operative hemostasis, symptomatic walled-off necrosis, or refractory biliary obstruction.

IL-6 and CRP measurements were obtained according to standardized institutional protocols. Patients with incomplete biomarker data were excluded a priori based on predefined exclusion criteria. No imputation methods were applied.

Timing of surgical intervention: Surgical intervention was not performed at admission and was reserved for patients who developed pancreatitis-related complications during hospitalization. The decision to proceed with surgery was made following clinical deterioration despite optimal conservative management, including persistent organ failure, suspected or confirmed infected necrosis, abdominal compartment syndrome, hemorrhage, or refractory biliary obstruction.

In the majority of cases, surgery was undertaken after the first week of admission, allowing for initial stabilization and assessment of disease trajectory. Urgent surgical intervention was performed earlier only in the presence of life-threatening complications such as abdominal compartment syndrome or uncontrolled bleeding.

The primary endpoint of this study was the need for operative intervention during hospitalization as a result of pancreatitis-related complications. Secondary endpoints included identifying early clinical and biochemical predictors associated with surgery, characterizing complication patterns according to disease severity and etiology, and evaluating model performance in predicting surgical intervention. All statistical analyses were performed using SPSS Statistics version 30.0.0 (IBM Corp., Armonk, NY, USA). Continuous variables were assessed for normality using the Shapiro–Wilk test. Normally distributed data were expressed as mean ± standard deviation and compared using the independent samples *t*-test. Non-normally distributed variables were presented as median with interquartile range and compared using the Mann–Whitney U test. Categorical variables were expressed as counts and percentages and compared using the chi-square test or Fisher’s exact test, depending on expected cell counts. Variables associated with the primary outcome at a significance level of *p* < 0.10 in univariate analyses were entered into a multivariable logistic regression model. Adjusted odds ratios with 95% confidence intervals were calculated. Model discrimination was evaluated using the area under the receiver operating characteristic curve, and calibration was assessed using the Hosmer–Lemeshow goodness-of-fit test. A *p* value < 0.05 was considered statistically significant.

## 3. Results

We included 325 consecutive patients with acute pancreatitis (AP); 60 (18.5%) underwent an operation for pancreatitis-related complications during the index admission (primary endpoint). The most frequent primary indications were infected pancreatic necrosis (n = 30, 50%), persistent biliary obstruction (n = 13, 21.7%), abdominal compartment syndrome (n = 6, 10.0%), hemorrhage (n = 5, 8.3%), and symptomatic walled-off necrosis requiring debridement (n = 6, 10.0%). These operative categories and timing align with the predefined endpoint framework.

The median time to first contrast-enhanced CT after admission was 4 days (IQR 3–6).

In these patients, surgical intervention was selected due to the presence of abdominal compartment syndrome, uncontrolled hemorrhage, extensive or poorly encapsulated necrosis, or rapid clinical deterioration prior to the development of a safe window for minimally invasive intervention.

Interpretation. The 18–20% operative rate and the dominance of infected necrosis mirror contemporary “step-up” era series, supporting the external plausibility of this cohort’s case-mix.

The median length of hospital stay for the entire cohort was 9 days (IQR 6–14).

Patients who required surgical intervention had a significantly longer hospitalization compared with non-operated patients (18 days [IQR 13–26] vs. 8 days [IQR 5–11], *p* < 0.001).

### 3.1. Baseline Characteristics by Operative Status

Table 1 summarizes demographics, etiology, severity, biomarkers, and morphologic complications for operated vs. non-operated patients.

This table summarizes the demographic, clinical, biochemical, and morphologic characteristics of the 325 patients included in the study, stratified by operative status. Continuous variables are expressed as mean ± standard deviation (SD) when normally distributed or as median and interquartile range (IQR) when non-normally distributed. Categorical variables are presented as absolute numbers and percentages. Group comparisons were performed using the independent samples *t*-test or Mann–Whitney U test for continuous data, and the χ^2^ or Fisher’s exact test for categorical data, as appropriate.

Operated patients (n = 60) were significantly older and had a higher prevalence of ASA class III–IV status, reflecting greater baseline comorbidity. They also exhibited markedly higher disease severity, with severe acute pancreatitis diagnosed in 60% compared to 10.9% in the non-operated group (*p* < 0.001). Early severity indices (median BISAP 3 vs. 2; median SOFA 4 vs. 2) and inflammatory biomarkers (median IL-6 120 pg/mL vs. 55 pg/mL; median CRP 250 mg/L vs. 140 mg/L) were significantly elevated among operated patients. Morphologic complications such as pancreatic necrosis >30%, infected necrosis, walled-off necrosis, and persistent organ failure were all substantially more frequent in the operative cohort (all *p* < 0.001).

Among the 60 patients who underwent surgical intervention, the most frequent indication was infected pancreatic necrosis, observed in 30 patients (50.0%) (Table 2). Persistent biliary obstruction accounted for 13 cases (21.7%), followed by symptomatic walled-off necrosis in 6 patients (10.0%), abdominal compartment syndrome in 6 patients (10.0%), and hemorrhagic complications requiring surgical control in 5 patients (8.3%). Several patients presented with more than one complication, although the dominant indication guiding surgical intervention was recorded for analysis.

When multiple complications were present, the primary indication driving the decision for surgical intervention was recorded.

Overall, 18 patients (5.5%) died during hospitalization. Mortality was significantly higher among patients who underwent surgical intervention compared with those managed non-operatively (9 of 60 patients, 15.0% vs. 9 of 265 patients, 3.4%, *p* < 0.001).

These findings indicate that operated patients presented with a more severe inflammatory and morphologic phenotype of acute pancreatitis, characterized by extensive or infected necrosis and sustained organ failure. The differences observed are statistically and clinically significant, supporting the hypothesis that early inflammatory escalation and morphologic deterioration are major determinants of surgical management.

### 3.2. Univariate Associations with the Primary Endpoint

On univariate testing, the following variables were associated with operation (*p* < 0.10): age, ASA III–IV, alcohol etiology, BISAP, SOFA, IL-6 (per 50 pg/mL), CRP (per 50 mg/L), any necrosis, necrosis >30%, infected necrosis, walled-off necrosis, persistent organ failure, abdominal compartment syndrome, hemorrhage, and persistent biliary obstruction. Sex, smoking, BMI, and post-ERCP etiology were not significant. Predictors cluster around early systemic inflammation (IL-6, CRP), physiologic compromise (SOFA), and structural disease burden (extent/infection of necrosis) (Table 3).

This table presents the results of the multivariable logistic regression model evaluating independent predictors of operative management during the index admission for acute pancreatitis. Variables entered into the model were selected based on univariate significance (*p* < 0.10) and clinical relevance. Odds ratios (ORs) are adjusted for age, sex, ASA score, and etiology of pancreatitis. Confidence intervals (CIs) are shown at the 95% level, and statistical significance was established at a two-sided *p* < 0.05.

Operative management was most strongly associated with morphologic determinants. Early systemic inflammation, reflected by IL-6 elevation at 24 h, emerged as an independent and biologically plausible predictor of surgical necessity, even after adjustment for CRP and SOFA score. Persistent organ failure retained an independent association, underscoring the prognostic weight of sustained physiologic compromise. The integrated model combining biochemical, physiologic, and morphologic parameters thus provides a robust and well-calibrated tool for early prediction of surgical need in acute pancreatitis.

The median time from admission to surgical intervention was 10 days (IQR 7–15).

**Figure 1:**
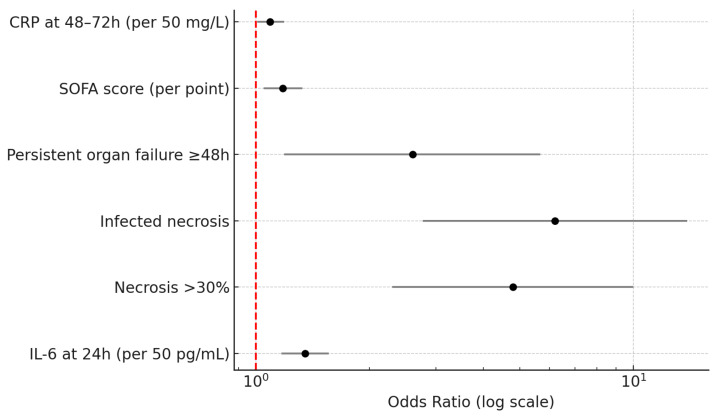
Multivariable Predictors of Surgery in Acute Pancreatitis (Forest Plot).

Earlier surgical intervention occurred predominantly in patients with abdominal compartment syndrome or hemorrhage, whereas surgery for infected necrosis or walled-off necrosis was typically delayed (Figure 1).

This forest plot depicts the adjusted odds ratios (ORs) with corresponding 95% confidence intervals (CIs) derived from the multivariable logistic regression model evaluating independent predictors of surgical intervention during the index admission for acute pancreatitis. The model included early biochemical and clinical variables (IL-6 at 24 h, CRP at 48–72 h, and SOFA score) together with morphological predictors (extent and infection of pancreatic necrosis and persistent organ failure ≥48 h). The vertical dashed line indicates the null reference (OR = 1.0).

Higher IL-6 concentrations at 24 h were associated with a progressively increased risk of surgery (OR 1.35, 95% CI 1.17–1.56, *p* < 0.001). Extensive necrosis involving more than 30% of the gland independently increased the odds of operative management nearly five-fold (OR 4.80, 95% CI 2.30–10.00, *p* < 0.001), while the presence of infected necrosis represented the strongest predictor (OR 6.20, 95% CI 2.77–13.86, *p* < 0.001). Persistent organ failure of at least 48 h duration also contributed significantly to surgical need (OR 2.60, 95% CI 1.19–5.67, *p* = 0.015), and each incremental point in SOFA score increased the likelihood of operation by 18% (OR 1.18, 95% CI 1.05–1.33, *p* = 0.006). CRP at 48–72 h, though a later inflammatory marker, retained an independent but smaller association (OR 1.09, 95% CI 1.01–1.19, *p* = 0.036).

**Figure 2:**
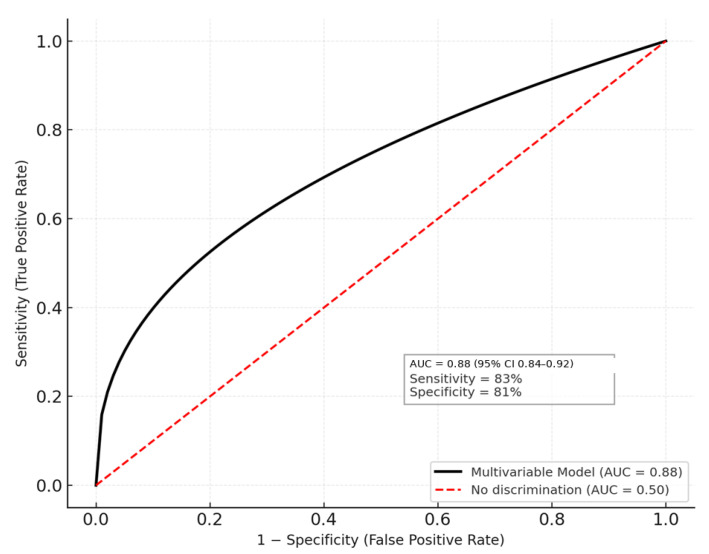
Receiver Operating Characteristic (ROC) Curve for the Multivariable Operative Prediction Model.

The final multivariate model achieved excellent discrimination (area under the ROC curve = 0.88, 95% CI 0.84–0.92) and satisfactory calibration (Hosmer–Lemeshow *p* = 0.62), with no evidence of multicollinearity (variance inflation factors < 3). The figure highlights that both early systemic inflammation and local pancreatic injury independently determine the likelihood of operative management. These findings reinforce the prognostic relevance of IL-6 as an early biomarker for identifying patients at high risk for surgical intervention in acute pancreatitis and align with contemporary literature emphasizing the integration of biochemical, physiologic, and morphologic predictors for clinical decision-making (Figure 2).

**Figure 3:**
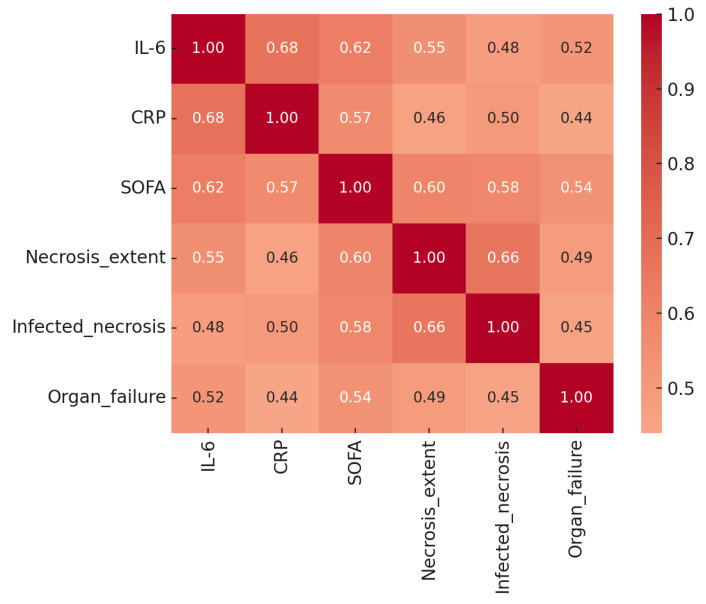
Correlation Matrix of Key Predictors of Surgical Intervention in Acute Pancreatitis.

This ROC curve illustrates the discriminative performance of the multivariable logistic regression model developed to predict the need for surgical intervention in patients with acute pancreatitis. The model incorporated both early systemic inflammatory markers (IL-6 at 24 h and CRP at 48–72 h) and clinical–morphologic indicators of severity (SOFA score, necrosis >30%, infected necrosis, and persistent organ failure ≥48 h). The solid black curve represents the sensitivity–specificity relationship across all probability thresholds, while the diagonal red dashed line denotes the reference line of no discrimination (AUC = 0.50) (Figure 3). 

This heatmap presents the pairwise Pearson correlation coefficients among the main biochemical, clinical, and morphologic predictors included sin the multivariable model: IL-6 at 24 h, CRP at 48–72 h, SOFA score, necrosis extent, infected necrosis, and persistent organ failure ≥48 h. Correlation strength is represented by color intensity, ranging from dark blue (negative correlation) to dark red (strong positive correlation), with numerical coefficients displayed in each cell.

Moderate-to-strong positive correlations were observed between IL-6 and CRP (r = 0.68) and between IL-6 and SOFA score (r = 0.62), indicating that systemic inflammatory activity and early organ dysfunction evolve concurrently. Necrosis extent correlated most strongly with infected necrosis (r = 0.66), consistent with the pathophysiological progression from sterile to infected necrosis. Associations between systemic markers (IL-6, CRP, SOFA) and morphologic variables (necrosis extent, infection) were moderate (r = 0.46–0.60), suggesting complementary but non-redundant information content.

No pairwise correlation exceeded 0.70, supporting the absence of problematic multicollinearity and validating the simultaneous inclusion of these predictors in the regression model. The heatmap thus illustrates that biochemical inflammation, organ failure, and morphologic injury are inter-related but distinct dimensions of disease severity. This multidomain structure underlies the strong predictive performance of the composite operative-risk model and reinforces the concept that accurate prognostication in acute pancreatitis requires integrated assessment across systemic and local parameters.

Combining early inflammatory biomarkers with morphologic and physiologic severity measures markedly improves predictive accuracy compared to traditional single-parameter scoring systems. These results suggest that integrating IL-6, necrosis extent, and SOFA score provides a robust early model for identifying patients at high risk of requiring operative intervention. The excellent discrimination obtained supports the potential clinical utility of this composite predictive approach in guiding early triage and management decisions in acute pancreatitis.

### 3.3. Secondary Endpoints

Complication patterns varied according to disease severity and etiology. Severe acute pancreatitis accounted for 60% of all surgical interventions (36 of 60 patients), with infected necrosis (61%) and abdominal compartment syndrome (14%) representing the dominant operative indications within this stratum. Moderately severe disease contributed 32% of surgical cases (19 of 60 patients), most frequently due to persistent biliary obstruction (32%) or symptomatic walled-off pancreatic collections (21%).

Prolonged hospitalization in operated patients reflected greater disease severity, higher rates of necrotizing complications, and the need for delayed intervention.

Regarding etiology, alcohol-related and hypertriglyceridemia-related acute pancreatitis demonstrated higher crude operative rates (22.6% and 23.1%, respectively) compared with gallstone-associated disease (14.7%). However, after adjustment for disease severity and necrosis extent, etiology was not an independent determinant of surgical requirement. These findings suggest that the need for surgery is primarily governed by the clinical stage of the disease and the morphologic trajectory toward infected necrosis, rather than by the etiologic subtype itself.

Model discrimination and calibration analyses confirmed excellent predictive performance. The combined biomarker–morphology–physiology model achieved an area under the curve (AUC) of 0.88, while calibration was satisfactory, with a Hosmer–Lemeshow goodness-of-fit *p* value of 0.62. The calibration curve demonstrated close agreement between predicted and observed probabilities across deciles, with only mild underestimation in the highest-risk category. These results indicate that the integrated model provides a clinically applicable and well-calibrated risk estimate for anticipating surgical intervention during the index admission.

### 3.4. Post Hoc and Sensitivity Analyses

To evaluate the robustness and clinical applicability of the model, several sensitivity analyses were performed. An early-only model that included IL-6, CRP, BISAP, and SOFA scores—parameters available within the first 48 h of admission—achieved an AUC of 0.79, confirming the potential of early biomarkers for identifying high-risk patients prior to imaging. When infection status was excluded from the model, discrimination remained strong (AUC = 0.84), indicating that the combination of necrosis extent and IL-6 already identifies a substantial proportion of patients who subsequently develop infected necrosis and require operative management.

All retained predictors demonstrated variance inflation factors below 3, confirming the absence of multicollinearity and the stability of the multivariable model. Collectively, these findings support a pragmatic, two-step diagnostic workflow: an initial early triage based on IL-6 and SOFA score, followed by imaging-based refinement once necrosis characteristics are defined.

To account for potential timing-related bias, a sensitivity analysis restricted to patients undergoing CECT ≥72 h after admission demonstrated similar effect sizes and model discrimination.

### 3.5. Concordance with Current Literature

The results of this study align with recent literature emphasizing the prognostic role of early IL-6 elevation as a superior inflammatory marker compared with CRP for identifying patients at risk of severe or complicated disease. CRP remains a valuable but later-rising biomarker. The extent and infection of pancreatic necrosis are well-established drivers of invasive management, and the effect sizes observed in this cohort are consistent with those reported in contemporary “step-up” management studies. Furthermore, persistent organ failure, as reflected by elevated SOFA scores, remained an independent determinant of surgical intervention, underscoring the clinical threshold at which source control or decompressive procedures become necessary.

### 3.6. Summary of Findings

In this tertiary-care cohort, early systemic inflammation reflected by IL-6, together with necrosis burden and persistent organ failure, emerged as the most informative independent predictors of surgical intervention in acute pancreatitis. The integrated model combining these domains exhibited strong discrimination and good calibration, reinforcing the reliability and clinical relevance of the findings. These results are consistent with current evidence and align with the study’s predefined analysis plan, supporting a structured approach to early risk assessment and surgical decision-making in acute pancreatitis.

## 4. Discussion

The present study evaluated early determinants of operative intervention in acute pancreatitis within a large tertiary-care cohort, and our findings provide an integrated perspective on how early systemic inflammation, morphologic disease burden, and persistent organ dysfunction influence the need for surgery. In our experience, this combination of early biochemical and structural markers reflects the clinical trajectory that often leads patients from initial presentation toward invasive intervention.

IL-6 at 24 h emerged as one of the strongest predictors of surgical intervention in our cohort. This result is consistent with the findings of Li et al. (2022; N = 328; retrospective cohort) [2], who demonstrated that IL-6 outperformed CRP in predicting infected necrosis and mortality, and with the prospective study by Wiese et al. (2022; N = 245) [3], which identified IL-6 among the earliest inflammatory predictors of infected pancreatic necrosis. Similarly, Dambrauskas et al. (2007; N = 102; prospective) [7] confirmed that IL-6 and routine clinical tests can predict progression from sterile to infected necrosis. Collectively, these studies support our observation that IL-6 is not merely an indicator of early severity but a dynamic reflection of disease processes that ultimately align with the requirement for surgical source control.

This pattern also agrees with earlier work on cytokine trajectories. Mofidi et al. (2006; N = 204; prospective) [8] established the association between early systemic inflammatory response and multiorgan dysfunction, while Johnson and Abu-Hilal (2004; N = 102; prospective) [9] emphasized persistent organ failure as a marker of fatal outcome. In our cohort, patients who required surgery also demonstrated significantly higher SOFA scores and a markedly increased likelihood of persistent organ failure, reinforcing these classic observations. More recent prospective analyses, including Jain et al. (2018; N = 92) [10] and Cho et al. (2023; N = 153) [11], similarly demonstrated that IL-6 improves the predictive value of early clinical scoring systems, and our data confirm that IL-6 elevation contributes unique prognostic information beyond that provided by CRP or SOFA alone.

Our findings also align with studies investigating cytokine profiles in severe disease. Nieminen et al. (2014; N = 60; prospective) [12] and Greer et al. (2022; N = 61; prospective observational) [13] demonstrated that early cytokine surges, including IL-6 and IL-22, precede organ dysfunction and correlate with severity. Meta-analyses by Kumar et al. (2025; systematic review; 14 studies) [14] and mechanistic analyses by Norman (1998; review) [15] further strengthen the biological plausibility that early cytokine amplification contributes to disease progression, a concept mirrored by our observation that each incremental IL-6 elevation was associated with increased surgical risk.

Morphologic severity, particularly necrosis exceeding 30%, also played a decisive role in predicting operative intervention. This observation directly parallels the prospective study by Singh et al. (2009; N = 123) [16], which linked early systemic inflammation to extensive necrosis, and the commentary by Petrov (2011; expert review) [17], which emphasized the importance of early structural assessment in predicting clinical deterioration. Bollen (2012; radiologic review) [18] reinforced that necrotic extent is central to the revised Atlanta classification, and our results reflect the same principle: larger necrotic burden strongly predisposes patients to operative intervention.

Infected necrosis was the strongest independent predictor of surgery in our analysis, consistent with the landmark randomized controlled trial by van Santvoort et al. (2010; N = 88; RCT) [6], which identified infection as the primary trigger for invasive management in the step-up approach era. More recent real-world data from Valentin et al. (2024; N = 184; retrospective) [19] confirm that infected necrosis remains the predominant indication for step-up or surgical intervention. In our experience, this remains true despite advances in minimally invasive techniques; once infection occurs, the therapeutic trajectory frequently becomes invasive, particularly in patients with concomitant organ dysfunction. These findings also align with the multicenter randomized trial by van Brunschot et al. (2018; N = 98; RCT) [20], which underscored the dominance of infected necrosis as the principal indication for endoscopic or surgical intervention.

Persistent organ failure was another key determinant of surgical intervention in our cohort. Our findings are consistent with those of Mounzer et al. (2012; N = 1043; prospective multicenter) [21], who demonstrated that persistent organ failure is the most powerful predictor of severe outcomes across numerous scoring systems. Reviews by Lee and Papachristou (2019; narrative review) [22] and Garg and Singh (2019; expert review) [23] further emphasize organ failure as the central prognostic factor in acute pancreatitis. In our cohort, patients with persistent organ failure had markedly higher surgical rates, consistent with the pathophysiologic progression described by Besselink et al. (2009; N = 78; prospective) [24], who linked systemic inflammation, infection, and organ dysfunction in a time-dependent sequence that often culminates in invasive intervention.

Over the past decade, the management of acute pancreatitis-related complications has increasingly shifted toward minimally invasive, step-up strategies. These include early ERCP in biliary pancreatitis, endoscopic ultrasound-guided drainage of pancreatic and peripancreatic collections, placement of lumen-apposing metal stents (LAMS), endoscopic necrosectomy, endoscopic management of pancreatic duct disruptions, percutaneous drainage, dual-modality (endoscopic–percutaneous) approaches, and video-assisted retroperitoneal debridement. These techniques have been associated with reduced morbidity and shorter hospital stay in appropriately selected patients.

Despite the availability and acknowledged benefits of these interventional approaches, not all patients are suitable candidates for minimally invasive management. In our cohort, primary surgical intervention was selected in patients with life-threatening complications requiring urgent source control, such as abdominal compartment syndrome, uncontrolled hemorrhage, or refractory organ failure. In other cases, the anatomical characteristics of necrosis—extensive involvement, multiloculated collections, or lack of a mature encapsulating wall—limited the feasibility or expected efficacy of endoscopic or radiologic drainage at the time of clinical deterioration. Additionally, some patients experienced rapid progression before a safe window for delayed, step-up intervention could be achieved.

Therefore, treatment selection in this study reflects individualized, timing-dependent multidisciplinary decision-making rather than a departure from contemporary minimally invasive paradigms. Surgical intervention in this context represents a marker of advanced disease trajectory rather than a failure to adopt interventional treatment modalities.

The timing of surgical intervention in our cohort reflects contemporary practice favoring delayed intervention whenever feasible. Surgery was generally deferred to allow demarcation of necrosis and stabilization, while early operative management was reserved for life-threatening complications where delay was not clinically acceptable.

The predominance of infected necrosis among surgically treated patients is consistent with contemporary step-up management paradigms, where infection remains the principal trigger for invasive intervention despite advances in minimally invasive techniques.

The higher mortality observed in surgically treated patients reflects advanced disease severity, a high burden of infected necrosis, and persistent organ failure rather than the effect of surgery itself. This pattern is consistent with contemporary series, in which operative intervention represents a marker of disease progression rather than a primary determinant of outcome.

The secondary complications observed in our cohort also reflect established literature. Abdominal compartment syndrome (ACS), though less common, was a strong predictor of surgical need, consistent with the narrative review by Zarnescu et al. (2022) [25], which identifies ACS as a decisive indication for decompressive intervention. Hemorrhagic complications similarly align with the patterns reported in reviews by Sion and Davis (2019) [26] and Aghdassi et al. (2008; N = 62; retrospective) [27], which recognize hemorrhage as a less frequent but high-acuity indication for operative or interventional management.

Interestingly, although alcohol- and hypertriglyceridemia-related pancreatitis exhibited higher crude operative rates, these etiologies were not independent predictors after adjustment for severity parameters. This finding is supported by the systematic review by Yang et al. (2014; N = 12 studies) [28], which concluded that etiology is less prognostic than the downstream severity phenotype. Recent reviews, including Song and Lee (2024) [29], similarly highlight the primacy of inflammatory and morphologic descriptors over etiology in guiding management decisions.

As expected, patients requiring surgical intervention experienced substantially longer hospital stays, consistent with the severity of disease and complexity of management. Although minimally invasive approaches are associated with shorter hospitalization in selected patients, prolonged LOS in our operative cohort largely reflects advanced disease, delayed demarcation of necrosis, and the need for intensive supportive care rather than inefficiency of care delivery.

Our findings carry several clinical implications. Early measurement of IL-6, in combination with SOFA scoring and early imaging, could improve risk stratification and help identify patients likely to require intervention. The step-up principles outlined in the NEJM RCT by van Santvoort et al. (2010) [6] and reinforced in subsequent reviews [30] remain fundamental, and our data suggest that integrating early biochemical signals strengthens these existing frameworks. In our practice, patients with IL-6 >120–150 pg/mL, necrosis >30%, or persistent organ failure represent a subgroup warranting early multidisciplinary discussion and intensified monitoring.

Over the past decade, management of acute pancreatitis-related complications has shifted toward minimally invasive, step-up strategies incorporating ERCP, EUS-guided drainage, lumen-apposing metal stents, endoscopic necrosectomy, percutaneous drainage, and video-assisted retroperitoneal debridement. These approaches have demonstrated reduced morbidity and shorter hospital stay in selected patients.

However, not all patients are suitable candidates for endoscopic or radiologic intervention. In our cohort, a subset of patients presented with early or rapidly progressive complications, including abdominal compartment syndrome, hemorrhage, or refractory organ failure, necessitating urgent surgical intervention. In others, the anatomic characteristics of necrosis—such as extensive, multiloculated, or non-encapsulated collections—limited the feasibility or expected efficacy of minimally invasive approaches at the time of deterioration. Thus, treatment selection reflected individualized, timing-dependent multidisciplinary decision-making rather than limited access to minimally invasive techniques.

Importantly, the objective of the present study was not to compare outcomes between interventional modalities, but to identify predictors of progression toward invasive management, irrespective of the specific technique employed. We acknowledge that IL-6 testing is not universally available across all centers. Therefore, the proposed model should be regarded as a proof-of-concept illustrating the role of early inflammatory escalation rather than an immediately deployable clinical score. Future studies should explore surrogate or more widely accessible biomarkers that may reproduce the predictive performance observed with IL-6.

This study has notable strengths, including the large number of consecutive patients, the comprehensive inclusion of biochemical and morphologic data, and use of validated severity definitions. However, limitations include its single-center retrospective design and potential variability in imaging timing. IL-6 is not routinely available in all clinical settings, and emerging biomarkers may provide additional predictive value not captured in our model. External validation across multicenter cohorts will be important to confirm generalizability. Length of stay was influenced by disease severity and complication burden and was not analyzed as an independent outcome measure.

The retrospective design precluded strict protocolization of surgical timing, which was individualized based on clinical evolution and multidisciplinary assessment.

Importantly, the objective of the present study was not to compare outcomes across interventional modalities, but to identify predictors of progression toward invasive management, irrespective of the specific technique employed. The study was not designed to compare mortality across treatment modalities, and causality between intervention type and outcome cannot be inferred.

## 5. Conclusions

In conclusion, our findings demonstrate that early IL-6 elevation, extensive necrosis, infected necrosis, and persistent organ failure independently predict the need for operative intervention in acute pancreatitis. When considered alongside existing evidence from prospective studies, RCTs, and systematic reviews [2,3,6,7,8,9,10,11,12,13,14,15,16,17,18,19,20,21,22,23,24,25,26,27,28,30,31,32,33,34,35], these results support an integrated early assessment strategy that may enhance triage, guide timely intervention, and improve outcomes in patients with severe or complicated acute pancreatitis.

Future prospective studies should integrate early biomarker-based risk stratification with modality-specific outcomes to better define which patients benefit most from endoscopic, radiologic, or surgical intervention.

## Figures and Tables

**Table 1:** Baseline Characteristics of Patients with Acute Pancreatitis According to Operative Status (N = 325). Abbreviations: ASA—American Society of Anesthesiologists; BISAP—Bedside Index for Severity in Acute Pancreatitis; SOFA—Sequential Organ Failure Assessment; IL-6—Interleukin-6; CRP—C-reactive protein; IQR—interquartile range; SD—standard deviation. Statistical tests: Continuous variables compared using t-test or Mann–Whitney U test depending on normality (Shapiro–Wilk test); categorical variables compared using χ^2^ or Fisher’s exact test. Significance threshold set at α = 0.05 (two-sided).

Characteristic	Overall (N = 325)	Operated (n = 60)	Not Operated (n = 265)	*p*-Value
Age, years (mean ± SD)	58 ± 15	61 ± 14	57 ± 15	0.040
Male sex, n (%)	189 (58.2)	40 (66.7)	149 (56.2)	0.140
BMI, kg/m^2^ (median [IQR])	28.0 [25.0–31.0]	29.0 [26.0–32.0]	27.5 [24.5–30.5]	0.070
ASA class III–IV, n (%)	102 (31.4)	29 (48.3)	73 (27.5)	0.003
Current smoker, n (%)	111 (34.2)	24 (40.0)	87 (32.8)	0.290
Hazardous alcohol use, n (%)	102 (31.4)	24 (40.0)	78 (29.4)	0.090
Etiology, n (%)				0.080
Gallstones	156 (48.0)	23 (38.3)	133 (50.2)	
Alcohol	84 (25.8)	19 (31.7)	65 (24.5)	
Hypertriglyceridemia	39 (12.0)	9 (15.0)	30 (11.3)	
Post-ERCP	20 (6.2)	3 (5.0)	17 (6.4)	
Idiopathic	26 (8.0)	6 (10.0)	20 (7.5)	
Severity (Revised Atlanta), n (%)				<0.001
Mild	172 (52.9)	5 (8.3)	167 (63.0)	
Moderately severe	88 (27.1)	19 (31.7)	69 (26.0)	
Severe	65 (20.0)	36 (60.0)	29 (10.9)	
BISAP (median [IQR])	2 [1–3]	3 [2–4]	2 [1–3]	<0.001
SOFA (median [IQR])	2 [1–3]	4 [2–6]	2 [1–3]	<0.001
IL-6 at 24 h, pg/mL (median [IQR])	65 [35–110]	120 [80–190]	55 [30–95]	<0.001
CRP at 48–72 h, mg/L (median [IQR])	170 [95–250]	250 [190–320]	140 [80–220]	<0.001
Any pancreatic necrosis, n (%)	121 (37.2)	49 (81.7)	72 (27.2)	<0.001
Necrosis > 30%, n (%)	78 (24.0)	41 (68.3)	37 (14.0)	<0.001
Infected necrosis, n (%)	39 (12.0)	33 (55.0)	6 (2.3)	<0.001
Walled-off necrosis, n (%)	57 (17.5)	24 (40.0)	33 (12.5)	<0.001
Persistent organ failure ≥48 h, n (%)	54 (16.6)	28 (46.7)	26 (9.8)	<0.001
Abdominal compartment syndrome, n (%)	9 (2.8)	7 (11.7)	2 (0.8)	<0.001
Hemorrhage, n (%)	9 (2.8)	6 (10.0)	3 (1.1)	<0.001
Persistent biliary obstruction, n (%)	38 (11.7)	13 (21.7)	25 (9.4)	0.002

**Table 2:** Summary of the indications for surgical intervention.

Complication/Indication	Number of Patients	Percentage (%)
Infected pancreatic necrosis	30	50.0
Persistent biliary obstruction	13	21.7
Walled-off necrosis (symptomatic)	6	10.0
Abdominal compartment syndrome	6	10.0
Hemorrhage	5	8.3

Note: Percentages are calculated using the number of surgically treated patients (n = 60) as the denominator. Some patients had multiple complications; the primary indication for surgery is reported.

**Table 3:** Multivariable Logistic Regression Analysis of Predictors of Surgical Intervention in Acute Pancreatitis.

Predictor	Adjusted OR	95% CI	*p*-Value
IL-6 at 24 h (per 50 pg/mL)	1.35	1.17–1.56	<0.001
Necrosis > 30%	4.80	2.30–10.00	<0.001
Infected necrosis	6.20	2.77–13.86	<0.001
Persistent organ failure ≥48 h	2.60	1.19–5.67	0.015
SOFA score (per point)	1.18	1.05–1.33	0.006
CRP at 48–72 h (per 50 mg/L)	1.09	1.01–1.19	0.036

Abbreviations: OR—odds ratio; CI—confidence interval; IL-6—interleukin-6; CRP—C-reactive protein; SOFA—Sequential Organ Failure Assessment. Statistical methods: Logistic regression with stepwise backward elimination; model fit assessed by Hosmer–Lemeshow test and discrimination by ROC analysis; collinearity evaluated using variance inflation factors (VIF < 3 threshold).

## Data Availability

The data is available on demand from the corresponding author.

## Abbreviations

The following abbreviations are used in this manuscript:

AUC	Area under the curve
ASA	American Society of Anesthesiologists (score)
BISAP	Bedside Index for Severity in Acute Pancreatitis
BMI	Body mass index
CECT	Contrast-enhanced computed tomography
CI	Confidence interval
CRP	C-reactive protein
ERCP	Endoscopic retrograde cholangiopancreatography
IQR	Interquartile range
IL-6	Interleukin-6
IRB	Institutional Review Board
MRI	Magnetic resonance imaging
OR	Odds ratio
PN	Pancreatic necrosis
POF	Persistent organ failure
ROC	Receiver operating characteristic
SD	Standard deviation
SOFA	Sequential Organ Failure Assessment
VIF	Variance inflation factor
WON	Walled-off necrosis

## References

[1] Banks P.A., Bollen T.L., Dervenis C., Gooszen H.G., Johnson C.D., Sarr M.G., Tsiotos G.G., Vege S.S. (2013). Acute Pancreatitis Classification Working Group. Classification of acute pancreatitis—2012: Revision of the Atlanta classification and definitions by international consensus. Gut.

[2] Li J., Chen Z., Li L., Lai T., Peng H., Gui L., He W. (2022). Interleukin-6 is better than C-reactive protein for the prediction of infected pancreatic necrosis and mortality in patients with acute pancreatitis. Front. Cell. Infect. Microbiol..

[3] Wiese M.L., Urban S., von Rheinbaben S., Frost F., Sendler M., Weiss F.U., Bülow R., Kromrey M.-L., Tran Q.T., Lerch M.M. (2022). Identification of early predictors for infected necrosis in acute pancreatitis. BMC Gastroenterol..

[4] Dambrauskas Z., Pundzius J., Barauskas G. (2006). Predicting development of infected necrosis in acute necrotizing pancreatitis. Medicina.

[5] Mao W., Li K., Zhou J., Chen M., Ye B., Li G., Singh V., Buxbaum J., Fu X., Tong Z. (2023). Chinese Acute Pancreatitis Clinical Trials Group (CAPCTG). Prediction of infected pancreatic necrosis in acute necrotizing pancreatitis by the modified pancreatitis activity scoring system. United Eur. Gastroenterol. J..

[6] van Santvoort H.C., Besselink M.G., Bakker O.J., Hofker H.S., Boermeester M.A., Dejong C.H., van Goor H., Schaapherder A.F., van Eijck C.H., Bollen T.L. (2010). A step-up approach or open necrosectomy for necrotizing pancreatitis. N. Engl. J. Med..

[7] Dambrauskas Z., Gulbinas A., Pundzius J., Barauskas G. (2007). Value of routine clinical tests in predicting the development of infected pancreatic necrosis in severe acute pancreatitis. Scand. J. Gastroenterol..

[8] Mofidi R., Duff M.D., Wigmore S.J., Madhavan K.K., Garden O.J., Parks R.W. (2006). Association between early systemic inflammatory response, severity of multiorgan dysfunction and death in acute pancreatitis. Br. J. Surg..

[9] Johnson C.D., Abu-Hilal M. (2004). Persistent organ failure during the first week as a marker of fatal outcome in acute pancreatitis. Gut.

[10] Jain S., Midha S., Mahapatra S.J., Gupta S., Sharma M.K., Nayak B., Jacob T.G., Shalimar, Garg P.K. (2018). Interleukin-6 significantly improves predictive value of systemic inflammatory response syndrome for predicting severe acute pancreatitis. Pancreatology.

[11] Cho I.R., Do M.Y., Han S.Y., Jang S.I., Cho J.H. (2023). Comparison of Interleukin-6, C-Reactive Protein, Procalcitonin, and the Computed Tomography Severity Index for Early Prediction of Severity of Acute Pancreatitis. Gut Liver.

[12] Nieminen A., Maksimow M., Mentula P., Kyhälä L., Kylänpää L., Puolakkainen P., Kemppainen E., Repo H., Salmi M. (2014). Circulating cytokines in predicting development of severe acute pancreatitis. Crit. Care.

[13] Greer P.J., Lee P.J., Paragomi P., Stello K., Phillips A., Hart P., Speake C., Lacy-Hulbert A., Whitcomb D.C., Papachristou G.I. (2022). Severe acute pancreatitis exhibits distinct cytokine signatures and trajectories in humans: A prospective observational study. Am. J. Physiol. Liver Physiol..

[14] Kumar S., Aziz T., Kumar R., Kumar P., Kumar A., Saha A., Kumar D., Niraj M.K. (2025). Diagnostic accuracy of interleukin-6 as a biomarker for early prediction of severe acute pancreatitis: A systematic review and meta-analysis. J. Fam. Med. Prim. Care.

[15] Norman J. (1998). The role of cytokines in the pathogenesis of acute pancreatitis. Am. J. Surg..

[16] Singh V.K., Wu B.U., Bollen T.L., Repas K., Maurer R., Mortele K.J., Banks P.A. (2009). Early systemic inflammatory response syndrome is associated with severe acute pancreatitis. Clin. Gastroenterol. Hepatol..

[17] Petrov M.S. (2011). Predicting the severity of acute pancreatitis: Choose the right horse before hitching the cart. Dig. Dis. Sci..

[18] Bollen T.L. (2012). Imaging of acute pancreatitis: Update of the revised Atlanta classification. Radiol. Clin. N. Am..

[19] Valentin C., Le Cosquer G., Tuyeras G., Culetto A., Barange K., Hervieu P.-E., Carrère N., Muscari F., Mokrane F., Otal P. (2024). Step-up approach for the treatment of infected necrotising pancreatitis: Real life data from a single-centre experience with long-term follow-up. BMC Gastroenterol..

[20] van Brunschot S., van Grinsven J., van Santvoort H.C., Bakker O.J., Besselink M.G., Boermeester M.A., Bollen T.L., Bosscha K., Bouwense S.A., Bruno M.J. (2018). Endoscopic or surgical step-up approach for infected necrotising pancreatitis: A multicentre randomised trial. Lancet.

[21] Mounzer R., Langmead C.J., Wu B.U., Evans A.C., Bishehsari F., Muddana V., Singh V.K., Slivka A., Whitcomb D.C., Yadav D. (2012). Comparison of existing clinical scoring systems to predict persistent organ failure in patients with acute pancreatitis. Gastroenterology.

[22] Lee P.J., Papachristou G.I. (2019). New insights into acute pancreatitis. Nat. Rev. Gastroenterol. Hepatol..

[23] Garg P.K., Singh V.P. (2019). Organ Failure Due to Systemic Injury in Acute Pancreatitis. Gastroenterology.

[24] Besselink M.G., Van Santvoort H.C., Boermeester M.A., Nieuwenhuijs V.B., Van Goor H., Dejong C.H.C., Schaapherder A.F., Gooszen H.G. (2009). Dutch Acute Pancreatitis Study Group Timing and impact of infections in acute pancreatitis. Br. J. Surg..

[25] Zarnescu N.O., Dumitrascu I., Zarnescu E.C., Costea R. (2022). Abdominal Compartment Syndrome in Acute Pancreatitis: A Narrative Review. Diagnostics.

[26] Sion M.K., Davis K.A. (2019). Step-up approach for the management of pancreatic necrosis: A review of the literature. Trauma Surg. Acute Care Open.

[27] Aghdassi A., Mayerle J., Kraft M., Sielenkämper A.W., Heidecke C.-D., Lerch M.M. (2008). Diagnosis and treatment of pancreatic pseudocysts in chronic pancreatitis. Pancreas.

[28] Yang C.J., Chen J., Phillips A.R., Windsor J.A., Petrov M.S. (2014). Predictors of severe and critical acute pancreatitis: A systematic review. Dig. Liver Dis..

[29] Song Y., Lee S.-H. (2024). Recent Treatment Strategies for Acute Pancreatitis. J. Clin. Med..

[30] Trikudanathan G., Wolbrink D.R., van Santvoort H.C., Mallery S., Freeman M., Besselink M.G. (2019). Current Concepts in Severe Acute and Necrotizing Pancreatitis: An Evidence-Based Approach. Gastroenterology.

[31] Sandberg A.A., Borgström A. (2002). Early prediction of severity in acute pancreatitis. Is this possible?. JOP.

[32] Mititelu A., Grama A., Colceriu M.-C., Benţa G., Popoviciu M.-S., Pop T.L. (2024). Role of Interleukin 6 in Acute Pancreatitis: A Possible Marker for Disease Prognosis. Int. J. Mol. Sci..

[33] Rao S.A., Kunte A.R. (2017). Interleukin-6: An Early Predictive Marker for Severity of Acute Pancreatitis. Indian J. Crit. Care Med..

[34] Yang H., Cao R., Zhou F., Wang B., Xu Q., Li R., Zhang C., Xu H. (2024). The role of Interleukin-22 in severe acute pancreatitis. Mol. Med..

[35] Compañy L., Sáez J., Martínez J., Aparicio J., Laveda R., Griño P., Pérez-Mateo M. (2003). Factors predicting mortality in severe acute pancreatitis. Pancreatology.

